# THE ASPREE STUDY: DEMENTIA DUE TO ALZHEIMER’S DISEASE OUTCOMES

**DOI:** 10.1093/geroni/igz038.2359

**Published:** 2019-11-08

**Authors:** Raj C Shah

**Affiliations:** Rush University, Chicago, Illinois, United States

## Abstract

In the ASPREE clinical trial, aspirin 100mg daily in health older adults did not delay onset of dementia, a pre-specified secondary outcome over a period of 5 years. We examine whether low-dose aspirin versus placebo is related to incident dementia due to Alzheimer’s disease. Older community-dwelling participants free of dementia, physical disability, and conditions requiring aspirin treatment were recruited (n=19,114). Participants were administered a cognitive test battery during follow-up and participants with suspected dementia underwent a more extensive dementia assessment. An expert international panel adjudicated dementia according to DSM-IV criteria, with sub-classification according to NIA-AA criteria. Over a median 4.7 years, 575 participants had a confirmed dementia diagnosis. In analysis of the 41% of cases classified as dementia probably due to Alzheimer’s disease, no difference in the incidence between the treatment arms was found (HR=0.96, 95% CI =0.74, 1.24). Plans for continued assessment of cognition in ASPREE-XT will be presented.

